# Digital approaches for predicting posttraumatic stress and resilience: promises, challenges, and future directions

**DOI:** 10.1192/j.eurpsy.2023.181

**Published:** 2023-07-19

**Authors:** K. Schultebraucks

**Affiliations:** Psychiatry, NYU Grossman School of Medicine, New York, United States

## Abstract

**Abstract:**

Digital technologies and advances in computational methods have become key drivers of innovation in many medical fields. In precision psychiatry, accurate and reliable measures of mental health are critical for informing patient care and clinical research. There has been growing concern over the limitations of traditional mental health assessments that are typically grounded in nosology defined by the DSM and are based on interviewer-led assessments or patient self-report questionnaires. Whereas such gold-standard clinical assessments can be cost-prohibitive, insensitive to change, and prone to subjective biases, the use of digital technologies provides an opportunity to improve the practical feasibility as well as the inter-rater and test-retest reliability of repeated mental health assessments. The key promise of this approach is to unlock the clinical potential of digital technologies in ways that foster research of high clinical relevance and impact on clinical care. I will discuss these promises and challenges for the future use of machine learning approaches for predicting and monitoring post-traumatic stress and resilience.

**Disclosure of Interest:**

None Declared

